# Supplementary material for: Spatial patterns in the contribution of biotic and abiotic factors to the population dynamics of three freshwater fish species
Source: PeerJ. 2022 Feb 23;10:e12857. doi: 10.7717/peerj.12857 (PMC8881916; doi:10.7717/peerj.12857)
Supplement: Supplemental Information 1 [file peerj-10-12857-s001.docx]

**Supplementary material**

**Appendix 1. Data summary statistics**


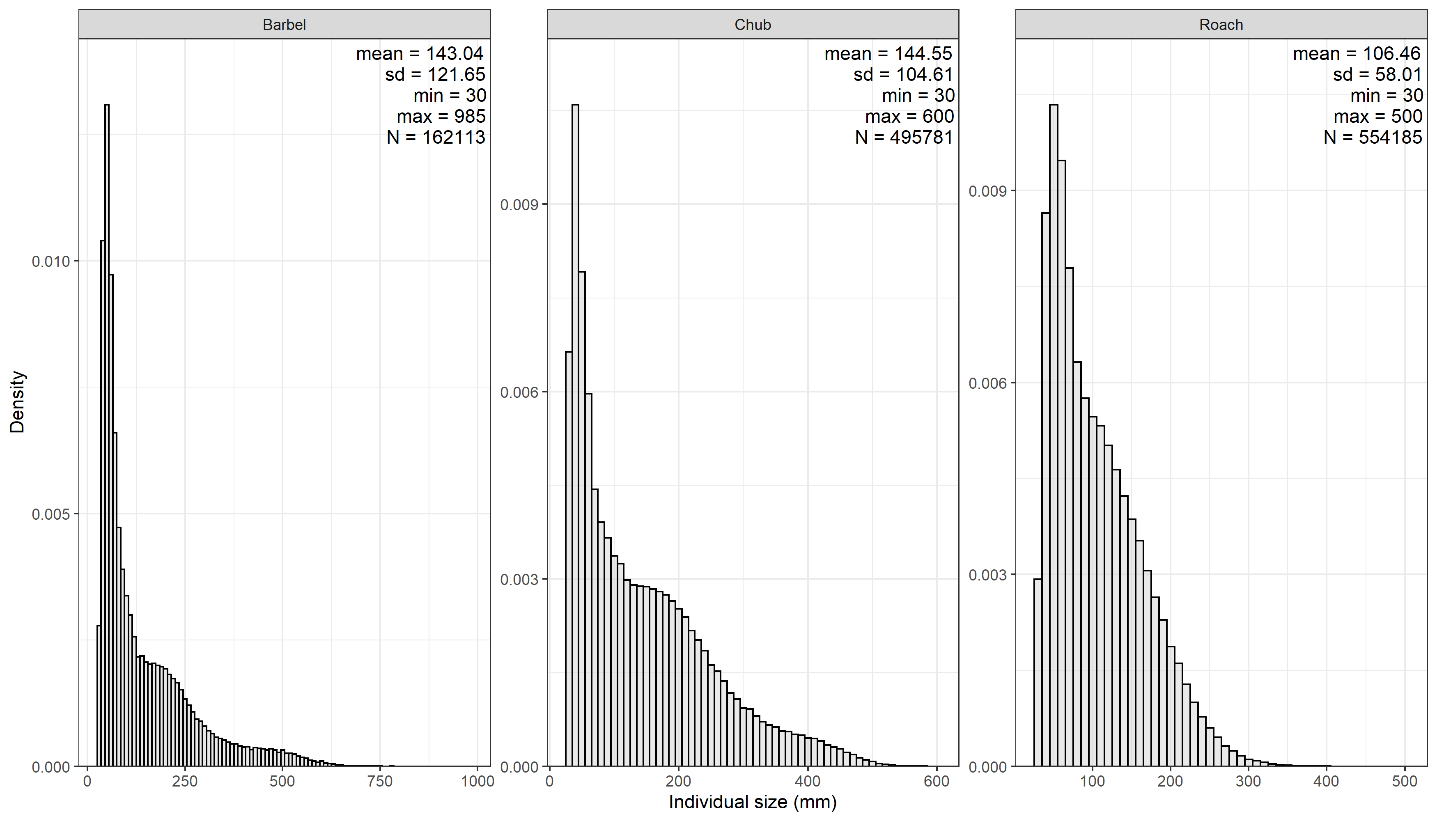


**Figure S1.** Full length-frequency histograms and summary of size data for the three studies species.


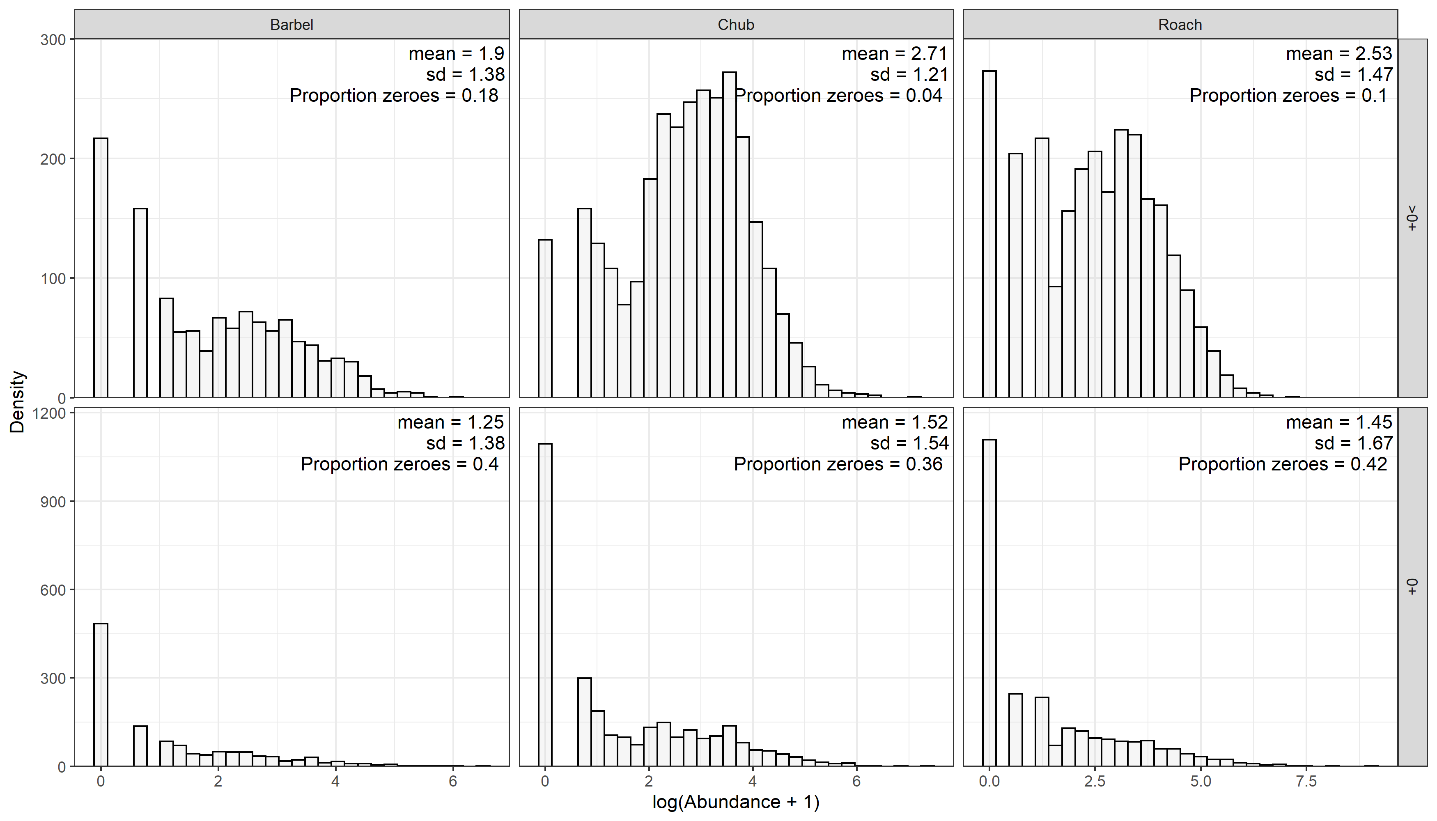


**Figure S2**. Raw abundance data and associated summary statistics.


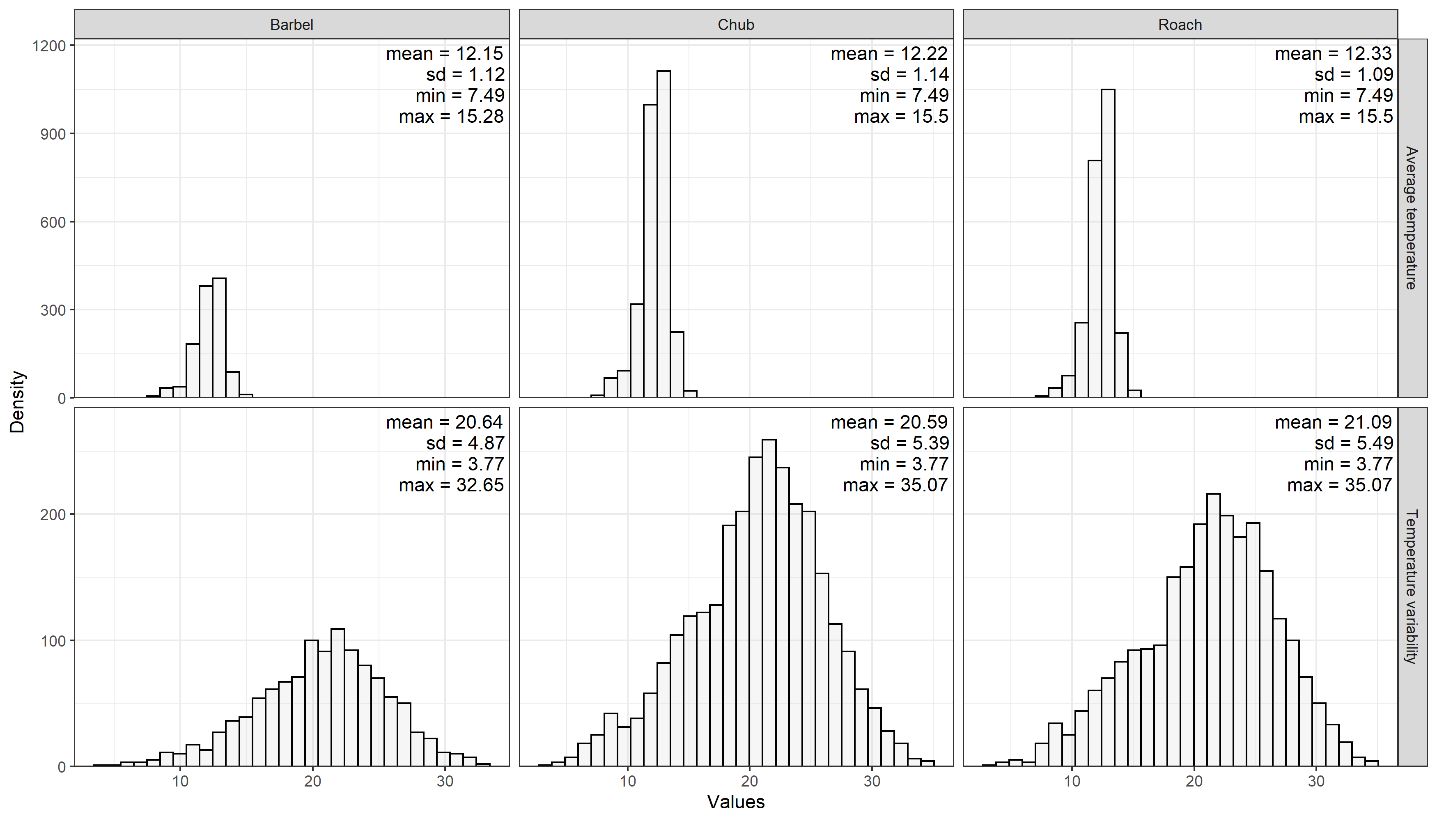


**Figure S3.** Raw temperature data and associated summary statistics.


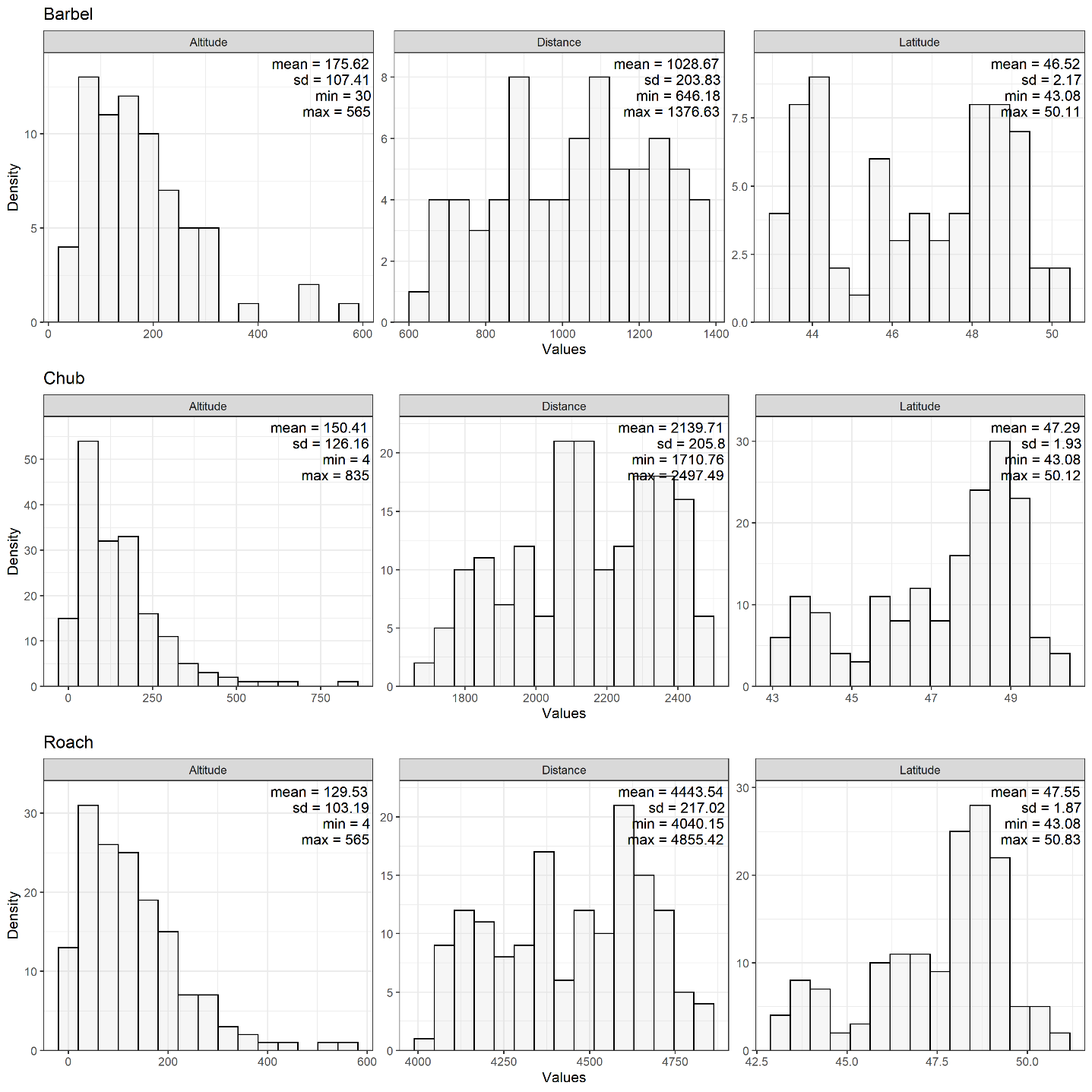


**Figure S4.** Spatial covariate data and associated summary statistics.
